# The patient-physician interactions as seen by undergraduate medical students

**DOI:** 10.1590/S1516-31802001000300002

**Published:** 2001-05-02

**Authors:** Leandro Yoshinobu Kiyohara, Lilian Kakumu Kayano, Marcelo Luís Teixeira Kobayashi, Mariana Sisto Alessi, Marina Uemori Yamamoto, Paulo Roberto Miziara Yunes, Rodrigo Rodrigues Pessoa, Rosana Mandelbaum, Silvio Tanaka Okubo, Thais Watanuki, Joaquim Edson Vieira

**Keywords:** Patient-physician interaction, Patient satisfaction, Teaching, Questionnaires, Relação médico-paciente, Satisfação, Ensino médico, Questionários

## Abstract

**CONTEXT:**

The interaction between a physician and his or her patient is complex and occurs by means of technical performance and through a personal relationship.

**OBJECTIVE:**

To assess the interaction between the medical professional and his or her patient with the participation of medical students assuming a role as observers and participants in a medical appointment in an outpatient office.

**DESIGN:**

Questionnaire interview study.

**SETTING:**

General Medicine outpatient offices, Hospital das Clínicas, Faculty of Medicine, University of São Paulo.

**PARTICIPANTS:**

Medical students performed an ethnographical technique of observation, following 199 outpatient medical appointments with Clinical Medicine Residents.

**MAIN MEASUREMENTS:**

A questionnaire filled out by observer students measured the physician's attitudes towards patients, as well as patients’ expectations regarding the appointment and his or her understanding after its completion.

**RESULTS:**

Patients showed higher enthusiasm after the appointment (4.47 ± 0.06 versus 2.62 ± 0.10) (mean ± SEM), as well as some negative remarks such as in relation to the waiting time. The time spent in the consultation was 24.66 ± 4.45 minutes (mean ± SEM) and the waiting time was 123.09 ± 4.91 minutes. The physician's written orientation was fairly well recalled by the patient when the doctor's letter could be previously understood.

**CONCLUSION:**

Patients benefit from physicians who keep the focus on them. In addition, this program stimulated the students for their accomplishment of the medical course.

## RESUMO

**CONTEXTO:**

A interação entre o médico e seu paciente em ambulatório.

**OBJETIVOS:**

observar a interação entre o médico e seu paciente, com a participação de estudantes de Medicina assumindo papel de observadores participantes em consultório.

**TIPO DE ESTUDO:**

Entrevista e questionário.

**LOCAL:**

Serviço de Clínica Geral, Ambulatório Geral e Didático, Hospital das Clínicas da Faculdade de Medicina da Universidade de São Paulo.

**PARTICIPANTES:**

Estudantes de medicina, no seu primeiro ano, realizaram uma técnica de observação etnográfica.

**VARIÁVEIS ESTUDADAS:**

Durante um ano, estudantes acompanharam 199 consultas ambulatoriais com Residentes da Clínica Médica. Usaram um questionário para observar a atitude do médico para com o paciente, como também as expectativas e o entendimento desse sobre a consulta.

**RESULTADOS:**

Pacientes demonstram estar mais animados após a consulta 4.47 (SEM 0.06) versus 2.62 (SEM 0.10) (depois x antes), apesar de algumas observações negativas como tempo de espera. O tempo de consulta foi de aproximadamente 24, 66 minutos (DP 4, 45) e o tempo de espera foi de 123, 09 minutos (DP 4, 91). A orientação por escrito, fornecida pelo médico, foi razoavelmente bem recordada quando a letra do médico podia ser entendida.

**CONCLUSÕES:**

Pacientes se beneficiam de médicos que lhes dão atenção mais do que nos aspectos fisiopatológicos de qualquer tipo de doença. Esse programa estimulou os estudantes no curso de Medicina, e aumentou o interesse por temas áridos como anatomia e bioquímica.

## INTRODUCTION

The interaction between the medical professional and his or her patient occurs by means of technical performance from the physician and through their personal relationship. While the first is entirely dependent on knowledge application from the doctor, increasing the benefits and reducing risks for this practice, the personal relationship is very complex. While participating in this momentary situation, the patient has his own social conventions, attitudes, expectations and needs, not necessarily exposed to the doctor, who has his or her own.^1^

Cognitive and clinical management skills as well as humanistic qualities and the ability to manage psychosocial aspects of illness have been indicated as important factors to be considered in a study of physician performance.^2^ In another report, the role models for medical education were investigated. The rating "excellent" was assigned to those devoting their time to teaching and conducting clinical rounds that included consultation tasks, outlining the importance of the doctor-patient relationship and giving attention to the psychosocial aspects of medicine.^3^

There appears to be a growing information content in the teaching of medicine, which is continually modifying the curriculum of medical schools. In spite of the various curricular arrangements of different schools that are directed towards the improvement of technical performance, there are some humanistic qualities that may be necessary. There is a consensus among doctors that some of these, like respect, compassion and integrity, are important in the physician's training. These qualities are strongly related to the doctor-patient interaction and can be better achieved when linked to the practice of medicine. When a professor attends a patient with respect and integrity, there is higher chance that the student will have the same type of behavior with his or her own patient.^4^

The early insertion of medical students into the practice of medicine has some advantages, such as facing up to the problems prompted by attitudes and values. In the doctor's office there will certainly be chances for such situations to arise, thus indicating an opportunity for the student to discuss the best ethical demeanor for the physician to adopt, in order to achieve the best possible result. On the other hand, it is understood that such a result will be the one that is closest to the patient's expectations, without reducing the importance of technical performance or moving away from the doctor's own ethical orientation.

It is not simple to measure the expectation that would ideally be communicated at the medical consultation. The field observation, however, can be adapted to this purpose. The techniques of ethnography can be quite rewarding for the acquisition and description of behaviors. During ethnographic observation four different roles can be assumed by the observer: 1 – complete participant, who is involved completely in the process, which may be affected by the observer's actions; 2 – participant and observer, who acts but also takes notes (research) of his or her own subjects, whose posture can be altered because their actions have been monitored; 3 – observer that participates, acting as an observer willing to intervene at any chance or invitation, which reduces the chance of modifying behaviors; and 4 – complete observer, who cannot even be seen in the process that is being studied.^5^

Adherence by the patient to the proposed treatment and the understanding of his or her health condition can be considered as the essential aspects of medical practice. Investigation of satisfaction in the doctor's office is one of the ways that can be used to improve these aspects. Some circumstances can be pointed to as being related to the patient's satisfaction: access to health services, outpatient service organization, treatment duration, doctor's competence and the patient's perception of it, clarity of information given by the doctor, doctor's behavior and patient's expectation. On the other hand, some factors do not seem to be linked to the patient's satisfaction, such as: type of payment, patient's clarity of communication, doctor's personality and patient's social and demographic characteristics or health condition.^6^

Some reports have shown up different aspects of this relationship: when patients had objective consultations (physical examination and history strictly focused on the problem presented by the patient) some of them appeared more satisfied. On the other hand, other patients were more comfortable when part of the consultation was spent in talking about trivial matters, including a " friendly" attitude by the doctor.^7,8^

Some authors suggest there are at least three main subjects to be addressed: i) the quality of attendance, ii) the access to the doctor, iii) the personal relationship with him or her. When using questionnaires to investigate this field, precision in the use of words is expected, so as not to give rise to contradictions. Also, the inclusion of open remarks may worsen data retrieval, but may even be essential for some results. The use of a five point scale (very poor, bad, regular, good, excellent) may be best for capturing patients’ opinions in this type of investigation.^9^

In this study, medical students took field observations of doctor-patient interactions during outpatient appointments. The students assumed the role of observer that participates and did a questionnaire intended to measure the doctor's attitudes to the patient, as well as the patient's expectations related to the appointment. The adoption of this questionnaire was proposed as complementary to the practice of free ethnographic annotation.

## METHODS

Students in their second semester (in their first year) from the University of São Paulo School of Medicine were allocated to a clinical medicine outpatient service, the Teaching Outpatient Service (AGD) at Hospital das Clínicas (Clinical Hospital), which is a university general hospital in the city of São Paulo, Brazil. They were allowed to follow a Resident during his or her clinical assignment, for four hours per week, with patients’ permission and in the knowledge they were students, and also subject to the Resident's willingness to receive them. We used a questionnaire with questions graded using a five point scale (very poor, bad, regular, good, excellent), some questions with yes or no answers and the retrieval of demographic data. The study protocol and questionnaire received approval from the Hospital Ethics Committee Board.

The patients were interviewed before the medical appointment, at the documentation desk, and invited to participate in the study, allowing one student to be inside the doctor's office. All patients were adults (45.05 years, SD 17.63), the female sex prevailed 2:1 (126 females, 65 males) and their educational level was somewhat low (2.18, SD 0.72), meaning that elementary schooling had been accomplished. Five questions were asked of the patient at this time. In the physician's office, another student took up the role of observer and was able to take notes regarding the medical consultation, including 6 items for the patient and 14 for the physician's attitudes. Immediately after leaving the consultation office, the patient was interviewed again by a third student with three additional questions pertinent to the results of the consultation. More specifically, the student had access to a chart with the doctor's orientation and asked the patient to repeat this to the best of their recall. The times spent waiting for the consultation and in it were recorded. In order not to lengthen the time spent by the patient during the scheduled visit to the hospital, the total filling-in time for the questionnaire was recorded, resulting in less than five minutes spent before the appointment and five minutes right after it.

*Statistical methods*. The data collected were analyzed using descriptive statistics, linear regression and the t-test, by means of a statistical software package, Sigma Stat for Windows Version 2.03 (SPSS Inc.). The inquiries about the understanding of the orientation given by the doctor, the understanding of his or her letter by the patient and the patient's satisfaction with the appointment were considered as dependent variables. Data were reported as means and standard deviations, and probability levels of less than 0.05 were considered significant.

## RESULTS

Doctors do not give written orientation often, regardless of whether the patient's appointment is a new one (37%) or a return visit (Table 1). Notwithstanding this, patients who were able to read these orientations also could repeat it during the recall test by students. The patient satisfaction was higher after the consultation. The total number of patients that allowed their appointments to be monitored by the students was 199. Some data were missed out, mainly regarding the time spent in the consultation (67 missing data items) and the satisfaction before the appointment (44 missing). Some patients did not comment on their educational level (58 missing).

**Table 1:** The attending Residents showed a strong tendency to not give written orientation to their patients, a tendency to ask questions requiring yes or no answers or open answers with the same frequency, and went straight to patient's complaints. 1 = very poor or rarely, 2, 3 and 4 as intermediate values, 5 = excellent or often

	1(very poor)	2	3	4	5 (excellent)	Missing
**Written orientation**	70	92	15	0	0	19
**Open questions**	12	25	45	58	54	2
**Questions with yes/no answers**	4	14	50	59	67	2
**Attention to complaints**	1	5	13	52	124	1

The doctor's orientation and letter and patient satisfaction did not show any significant correlation with all the other aspects of medical appointment. There was no related significance as to whether the doctor gives personal attention, allows the patient to interpret his or her own problems, or prepares for the physical examination. The attending Residents showed a tendency to greet the patients often (75%), to ask questions requiring yes/no answers or open answers with the same frequency, and went straight to the patients’ complaints (Table 1). They did not comment on the results of physical examinations, although when necessary they carefully explained laboratory tests, the medicines they suggested for patients to take, and they used a vocabulary appropriate to the patients’ understanding (Table 2).

**Table 2:** Residents did not comment on the results of physical examinations (P.E.), carefully explained the laboratory tests (71 situations where no laboratory tests needed), explained the medicines suggested to patients (42 situations where no medicines were prescribed) and used a vocabulary appropriate to patients' understanding very often. 1 = very poor or rarely, 2, 3 and 4 as intermediate values, 5 = excellent or often

	1 (very poor)	2	3	4	5 (excellent)	Missing	Not necessary
**Comments upon P.E.**	40	15	30	21	31	59	
**Explained Lab Tests**	9	14	19	32	34	17	71
**Explained medicines**	7	11	25	40	61	10	42
**Used fair vocabulary**	1	5	23	50	115	2	

**Figure 1:**
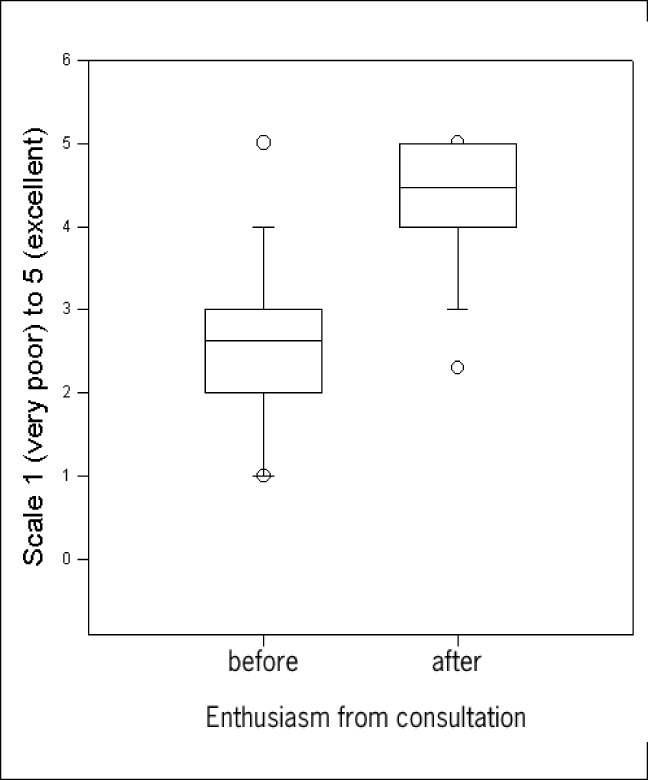
Enthusiasm before (2.62 [SEM 0.10]) and after (4.47 [SEM 0.06]) the doctor's appointment. Patients were shown to be happier right after the appointment (t-test, Mann-Whitney Rank Sum test, P < 0.001).

As expected, the patients did not show willingness in receiving prescriptions or laboratory tests, and they readily received appropriate explanations regarding their clinical declarations in the consultation (Table 3). Patients showed themselves to be happier right after the appointment (Figure 1). Enthusiasm was different before (2.62, SEM 0.10) and after (4.47, SEM 0.06) the doctor's appointment (t-test, Mann-Whitney Rank Sum test, P < 0.001), although the vast majority of comments were negative in remarks made about some matters like waiting time. The time spent in the consultation was 24.66 minutes (SEM 4.45) and the waiting time was 123.09 minutes (SEM 4.91).

**Table 3:** Patients did not show willingness to receive prescriptions or laboratory tests, and were ready to receive appropriate explanations regarding their clinical declarations in the consultation. 1 = very poor or rarely, 2, 3 and 4 as intermediate values, 5 = excellent or often

	1(rarely)	2	3	4	5 (often)	Missing
**Wanted prescription**	22	133	8	3	4	26
**Wanted Lab tests**	20	127	10	6	3	29
**Explanations from Doctors**	2	12	29	55	95	3

Patients who received a written orientation after the consultation were able to repeat it when they were able to understand the doctor's writing (linear regression, r = 0.404, a = 1.00). There was no correlation with the patients’ educational level, although those holding graduate degrees were very few among this observed population (5%).

## DISCUSSION

This study has pointed out the low readiness among doctors to give written orientation and the difficulties in understanding it, related to the letter format. Nevertheless, the satisfaction was higher after the medical appointment, even with a waiting time of almost two hours. It is important to emphasize the special features of this observation made by students who were perhaps not used to clinical practice, which could be a good point. Also, this was a narrow field represented by only one period per week in one hospital, which could be a negative point.

Doctors habitually have a more negative view of the consultation than the patients, including aspects of the ability to assess patients, to offer explanations and advice on treatment and to allow them to express their feelings during the consultation process.^10^ This impression can be confirmed and corroborated by the finding that physicians have a weak awareness of their patients’ responses, taking these as more negative than the patients themselves actually think.^11^ In addition, the imaginative and experiential responses of patients to medical explanations is a poorly explored area of medical study. When doctors try to detach their patients from this ability, confusion is likely and breakdowns in communication can occur.^12^ Finally, patient compliance increases when the drug instructions are well understood, but not necessarily correlated to satisfaction with communication.^13^ These previous reports suggest that the patient-physician interactions are not only related to the satisfaction during the visit, already complex, but also to the treatment compliance, another large and complex area. Both are crucial to any successful healthcare performance.

The finding in this report that our doctors do not give written explanations can be related to the supposed low educational level of these patients, most of whom only attended elementary school. However, given the previously reported impression of deeper negative feelings from physicians to patients, it is possible to consider this practice as being related to physicians’ impressions of low capacity among patients. Patients comply better with the proposed therapy when they understand the doctor's instruction. Since doctors were uneasy about writing them down, changing this habit would allow a better chance of improving the patient compliance with their care and therapies even more. Patients who ask questions about medications are seen in a positive light, rated as more interested in their own physical status.^14^ However, this capacity should not be taken for granted for all patients, and doctors who do not have such feedback may think of this as a feeling of inhibition caused by the patient's previous experiences.

The attending physicians during this study were first-year Residents. Teaching and attending patients are important aspects of creating role models of excellency for physician profiles.^3^ It is interesting to emphasize the hidden aspects of the patient-physician relationship uncovered by our new findings. Low willingness by physicians or a tendency not to greet patients should be considered as possibly resulting in a feedback of low expectations for the next or forthcoming scheduled visits. On the other hand, physicians’ careful attention to the explanation of laboratory tests and medicines, added to an ability to seek out adequate vocabulary, are all well developed techniques that help patients to become attuned to the proposed therapies.

Another aspect evoked by the students who participated was their upraised interest in the medicine course during the accomplishment of this project. The needs in medical education are not always clear, and sometimes students fall back into some idleness, as the course tackles uninteresting subjects. Although improving students’ interest was not the intention in this project, but rather it sought to facilitate familiarity with the scientific method and a capacity for writing such reports, their interest for different themes did improve during this experience, even biochemistry and anatomy. Since this was not the scope of the investigation, and we only have the reports of ten students out of almost two hundred, we are currently testing this approach with the new 2000 class at the USP School of Medicine (FMUSP).

Giving patients the possibility of talking after the appointment made them aware of some "consumer rights" as pointed out by some. The time spent waiting for the doctor to arrive or call the patients into the office was by far the most criticized aspect. Although the outpatient schedule considers the patient to be at the hospital at least half an hour before the appointment, the waiting time turned out to be far longer than expected, even by the Residents. We do not know what the main reason for this is, but it seems to be a deep-rooted problem for the hospital appointment books. There is already available a toll-free phone line for patients to confirm their appointments and program new ones, but perhaps this toll-free number has not been properly disclosed, or provision of the service is not enough yet (busy lines), or patients have not become used to it, showing a preference for coming early. Students considered the suggestion of having an auditor or a consumer rights assistant, with the setting up of a service where patients could leave their comments. It will be necessary to take into account the costs of new position of this nature, but perhaps the satisfaction would increase and a tighter schedule would make room for even more patient assistance.

## Conclusion

The use of questionnaires as ethnographic tools, putting students as observers into a physician's office, seems to be easy and accurate. This instrument could reveal physicians’ practices and their possible influence over patients’ attitudes. Patients were shown to have better compliance with doctors’ orientation when they had it written down, a practice that should be emphasized among doctors on duty.
